# Serum Growth Differentiation Factor 15 is Negatively Associated with Leukocyte Telomere Length

**DOI:** 10.1016/j.jnha.2025.100493

**Published:** 2025-02-03

**Authors:** Jie Yu, Yiwen Liu, Huabing Zhang, Fan Ping, Wei Li, Lingling Xu, Yuxiu Li

**Affiliations:** Department of Endocrinology, Key Laboratory of Endocrinology of National Health Commission, Peking Union Medical College Hospital, Chinese Academy of Medical Sciences & Peking Union Medical College, Beijing 100730

**Keywords:** Growth differentiation factor 15, Leukocyte telomere length, Mitochondrial DNA copy number, Aging

## Abstract

**Background:**

Telomere length(TL)and mitochondrial DNA copy number(mtDNAcn) are classic biomarker of aging. Recently, growth differentiation factor 15(GDF15) has attracted considerable attention as a vital component in the aging process.

**Methods:**

The present study aimed to study the relationship between GDF15 and telomere length and mtDNAcn.This was a cross-sectional analysis nested in a longitudinal cohort study conducted in Changping District, Beijing, from 2014 to 2021. Serum GDF15,leukocyte lelomere length(LTL) and mtDNAcn were determined in 802 subjects.LTL and mtDNAcn was quantified by real-time PCR assay. Multivariate linear regression and restricted cubic spline diagram were used for statistical analysis.

**Results:**

Subjects with higher GDF15 were older,had larger waist circumference, higher systolic blood pressure and glycated hemoglobin A1c (HbA1c),shorter LTL and tended to had less mtDNAcn. In correlation analysis, GDF15 was positively correlated with age, while LTL and mtDNAcn were negatively correlated with age.After adjusting for confounding factors,GDF15 was negatively associated with LTL (β = −0.120, 95%CI [−0.197, −0.042], p = 0.003) and the association was linear(p for nonlinear = 0.645), while the negative association between GDF15 and mtDNAcn did not reach significance.In the stratified analyses,the negative associations between GDF15 and LTL were more prominent in women, overweight individuals, or subjects with abnormal glucose tolerance (AGT), but similar results were observed in younger and older subjects.

**Conclusions:**

This study found a linear negative association between GDF 15 and LTL,which was more prominent in women, overweight or AGT subjects.These results supported that GDF15 might be a reliable biomarker of aging.

## 1 Introduction

Telomere is a small DNA-protein complex present at the end of linear chromosomes in eukaryotic cells, which consists of highly repetitive sequences of simple DNA, usually TTAGGG repeats [1]. The role of telomere is to protect the ends of chromosomes and maintain their integrity and stability.Telomere attrition is an important hallmark of aging [2].The measurement of blood telomere length(TL) has been recognized as a clinical indicator for assessing the risk of aging and age-related diseases [3].Genomic instability is another hallmark of aging, including nuclear DNA and mitochondrial DNA(mtDNA) [2]. MtDNA is the genetic material in mitochondria and number of mtDNA copies per cell (mtDNAcn) reflects mitochondrial health [4].MtDNAcn has been associated with aging and age related diseases [5,6].

Growth differentiation factor 15(GDF15) is a member of the transforming growth factor-β(TGF-β) superfamily, primarily involved in regulating cellular processes such as proliferation, differentiation, and apoptosis. It plays a pivotal role in regulating various biological effects such as tumorigenesis, inflammatory responses, and tissue injury [7]. Recently, GDF15 has gained significant attention as a crucial factor in the aging process, emerging as one of the most upregulated proteins with age and exhibiting a strong association with numerous age-related diseases [8,9].Furthermore,in a large population-based study, GDF15 was identified as one of the five senescence-associated secretory phenotype (SASP) proteins most strongly associated with increased risk of death [10].

Currently, the direct relationship between GDF15 and telomere length remains an area of ongoing research with limited definitive conclusions. However, recent studies have hinted at potential associations or correlations that warrant further investigation.A cross-sectional study included 120 healthy male individuals found that GDF15 showed a statistically significant linear and negative relationship with telomere length,PBMC telomerase activity, and telomerase reverse transcriptase(hTERT) mRNA after adjusting for confounders [11]. Moreover,a recent study found patients with scleroderma-associated interstitial lung disease have increased expression of GDF15 as well as shorter TL in lung tissue [12].However,another study including 169 healthy volunteers did not found associations between GDF15 with telomerase [13].

On the other hand,the direct relationship between GDF15 and mitochondrial DNA (mtDNA) copy number is also not well-established in current scientific literature.Recent studies have suggested a potential role for GDF15 as a biomarker for mitochondrial diseases [14,15]. Elevated levels of GDF15 have been observed in patients with mitochondrial diseases, and its levels have been correlated with disease severity [16].A study found a significant positive correlation between circulating cell-free mitochondrial DNA (ccfmtDNA) levels and GDF-15 concentrations in cerebrospinal fluid (CSF) of 25 patients with mitochondrial diseases [17].However, no previous studies have explore the relationship between GDF15 and mtDNA copy number in peripheral blood.

Given the small sample size and the inconsistent results in these studies, we aim to explore the relationship between GDF15, telomere length, and mitochondrial DNA copy number in a larger sample of individuals with multiple metabolic diseases.

## 2 Study design

The current analysis was based on a dynamic prospective longitudinal cohort study conducted in Changping District, Beijing, from 2014 to 2021.The baseline enrollment began in 2014, and since then baseline subjects would be followed up every 1-3 years, while new subjects would be enrolled at the same time and followed up regularly. This cohort included individuals from general population aged 18-80 years, with the aim of analyzing risk factors for aging and age-related diseases. The baseline enrollment in 2014 included 599 subjects described in detail previously [18], and 663 new subjects were enrolled during the follow-up, resulting in a total of 1262 subjects.

All participants completed a questionnaire administered by well-trained interviewers about history of chronic diseases and demographic and lifestyle characteristics. Fasting blood samples were collected, and all individuals underwent a physical examination. Additionally, after an overnight fast (8-12 h), all subjects (including subjects enrolled in 2014 and for new subjects) underwent an oral glucose tolerance test (OGTT), during which blood samples were taken at 0, 30, 60 and 120 min. Glucose tolerance status was classified according to the 1999 WHO criteria and glycated hemoglobin A1c (HbA1c) values from the 2001 America Diabetes Association criteria.

We excluded the following subjects: 186 subjects without blood samples, 182 subjects without leukocyte telomere length data, 27 subjects without mitochondrial DNA copy number data, 41 subjects without serum assay data, and 24 subjects with missing gender and age data. Finally, 802 subjects were included in this study. The Ethics Committee of Peking Union Medical College Hospital approved the study protocol, and all subjects signed written informed consent forms.

## 3 Methods

### 3.1 Anthropometric measurements

Weight (Wt) and height (Ht) were meticulously measured while the subjects wore light clothing and were barefoot to ensure accuracy. Specifically, height was recorded using a stadiometer, and weight was measured using a calibrated scale. The body mass index (BMI) was then calculated by dividing the weight in kilograms by the square of the height in meters.

Waist circumference (WC) was measured at the midpoint between the iliac crest (the top of the hip bone) and the costal margin (the lower edge of the rib cage), a standard approach to assessing abdominal obesity. A flexible tape measure was snugly wrapped around the subject's torso at this specified location, ensuring that the tape was parallel to the floor and not too tight or too loose to avoid inaccuracies.

Blood pressure (BP) readings were taken on the left arm using a standard mercury sphygmomanometer, which is recognized for its reliability and precision. Prior to measurement, subjects were instructed to sit quietly in a relaxed position for at least five minutes to ensure that their blood pressure was at a resting level. The cuff was placed around the upper arm, and the sphygmomanometer was adjusted to inflate the cuff until the artery was completely occluded. The cuff was then slowly deflated, and the first and fifth Korotkoff sounds were noted to determine the systolic and diastolic blood pressures, respectively. Each blood pressure measurement was performed twice by the same surveyor to minimize variability, and the average of the two readings was recorded for analysis.

### 3.2 Biochemical tests

Blood samples were prepared for immediate analysis or stored at −80 °C for further analysis. Serum glucose was tested in a glucose oxidase assay. Glycated hemoglobin A1c (HbA1c) was tested by high-performance liquid chromatography. Low-density lipoprotein cholesterol (LDL-C), triglyceride (TG), Total cholesterol (TC), high-density lipoprotein cholesterol (HDL-C), creatinine (Cr), and uric acid (UA) levels were measured by an automated analyzer. Insulin was measured by a chemiluminescence enzyme immunoassay.

For the measurement of growth differentiation factor 15 (GDF 15), serum was obtained from fasting blood samples to avoid any potential confounding effects of recent food intake. The levels of GDF15 were measured using the Luminex Assay Human Premixed Multi-Analyte Kit, following the manufacturer’s instructions provided by R&D Systems (Minneapolis, MN, USA). This kit utilizes a bead-based multiplexing technology, allowing for the simultaneous measurement of multiple analytes in a single sample, thus enhancing the efficiency and accuracy of the assay.

### 3.3 Measurement of LTL and mtDNAcn in peripheral blood

The measurement of LTL has been described in detail previously [18]. All blood samples were kept in a −80 °C refrigerator until they underwent testing. Genomic DNA from leukocytes in the peripheral blood samples was extracted utilizing the QIAamp DNA Blood Midi Kit (Qiagen, Hilden, Germany). Subsequently, the purified DNA samples were diluted and their concentrations were measured using a NanoDrop 1000 spectrophotometer (Thermo Fisher Scientific,Wilmington, DE, USA). The length of the telomeres was calculated as the ratio of the telomere repeat copy number to the single copy number (T/S), employing the innovative monochrome multiplex quantitative PCR protocol outlined by Cawthon [19],and the SYBR Green assays were employed. The within-plate and between-plate CVs were 18% and 7%, respectively.

The mtDNAcn measurement has also been described in detail previously [20]. Briefly, the relative mtDNAcn was measured by real-time PCR and corrected by the simultaneous measurement of nuclear DNA. The average intra-assay and interassay CVs were 4.2% (range, 1.6%–9.8%) and 4.6% (range, 0.9%–7.8%), respectively.

Since LTL and mtDNAcn were evaluated in different batches in 2014 and 2023, our current analysis aimed to minimize the potential influence of batch variation on measurements across years. To achieve this, we applied a log transformation to LTL and mtDNAcn data and subsequently standardized them by calculating a z score for each, comparing them to the respective means from 2014 and 2023 [21,22].

### 3.4 Statistical analysis

Quantitative variables with a normal distribution are presented as the means ± standard deviations (SDs). Nonnormally distributed parameters were transformed, and data that could not be normally distributed after conversion are presented as the median (interquartile range). Categorical data are presented as ratios or percentages. Descriptive statistics of population characteristics were calculated based on data grouped by GDF 15 levels. The chi-square test was used to compare categorical variables, and continuous variables were compared using independent sample t tests or the Mann-Whitney U test.

Firstly, Spearman’s correlation was performed to test the relationship between age and LTL,mtDNAcn as well as GDF 15. Secondly, multivariate linear regression was used to calculate β values of LTL and mtDNAcn with change of GDF 15. The model 1 was not adjusted,model 2 was adjusted for age,sex,BMI,WC,SBP,TG,HDL-C,UA and glycated hemoglobin A1c(HbA1c). Then, the associations between levels of GDF 15 and LTL and MtDNAcn were evaluated on a continuous scale with restricted cubic spline curves with 4 knots and the model was adjusted for confounding factors as model 2.

Subgroup analyses were conducted to compare effect sizes across different age groups (younger versus older), as well as by gender (male versus female), BMI categories (BMI <24 versus 24 ≤ BMI＜28 versus BMI ≥ 28), and glucose tolerance status (NGT versus DM/pre-DM).

All p-values are two-sided, and *P* < 0.05 indicated statistical significance. All statistical analyses were performed using SPSS, version 25.0 (IBM Corp., Chicago, IL, USA) and R version 3.6.3.

## 4 Results

**Fig. 1:**
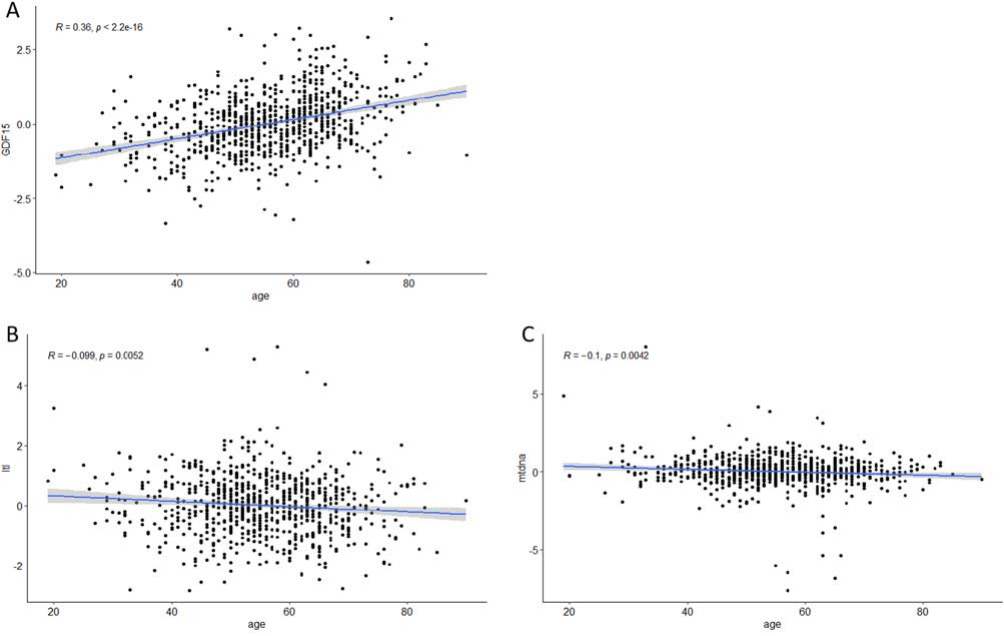
The correlations of GDF15,LTL and mtDNAcn and age. (A)GDF15-Age; (B)LTL-Age;(C)mtDNAcn-Age.

Baseline characteristics of the study population are presented in Table 1. Subjects with higher GDF15 were older,had larger WC, had higher SBP and glycated hemoglobin A1c (HbA1c). Subjects with higher GDF15 also had shorter LTL and tended to had less mtDNAcn. In correlation analysis,GDF15 was positively correlated with age, while LTL and mtDNAcn were negatively correlated with age(Fig. 1).

**Table 1:** Baseline characteristics of the study population.

	Total	Lower GDF15	Higher GDF15	p value
Number(n)	802	401	401	NA
Male(n, %)	280(34.9)	129(32.1)	151(37.6)	0.103
Age(years)	55.4 ± 11.0	52.2 ± 10.7	58.5 ± 10.4	<0.001*
BMI(kg/m^2^)	26.2 ± 3.7	26.2 ± 3.9	26.0 ± 3.6	0.47
WC(cm)	89.1 ± 11.0	87.6 ± 10.0	90.5 ± 11.7	<0.001*
SBP(mmHg)	129 ± 18	127 ± 18	132 ± 17	<0.001*
HbA1c(%)	6.2 ± 1.3	5.8 ± 1.0	6.5 ± 1.5	<0.001*
LgTG(mmol/L)	0.17 ± 0.27	0.17 ± 0.26	0.17 ± 0.27	0.938
HDL-C(mmol/L)	1.28 ± 0.37	1.30 ± 0.43	1.26 ± 0.29	0.157
UA(μmol/L)	294 ± 82	291 ± 75	296 ± 89	0.381
z-LgGDF15	0 ± 1	-0.76 ± 0.60	0.76 ± 0.67	<0.001*
z-LgLTL	0 ± 1	0.08 ± 1.02	-0.08 ± 0.96	0.011*
z-LgmtDNAcn	0 ± 1	0.06 ± 0.97	-0.06 ± 1.02	0.066

BMI:body mass index;WC:waist circumference;SBP:systolic blood pressure; HbA1c:glycated hemoglobin A1c;TG:triglyceride;HDL-C:high-density lipoprotein cholesterol;UA:uric acid;GDF15:growth differentiation factor 15;LTL:leukocyte lelomere length;mtDNAcn:mitochondrial DNA copy number.

* p < 0.05 indicates significance.

Table 2 shows the associations of GDF15 with LTL and mtDNAcn. After adjusting for confounding factors, GDF15 was negatively associated with LTL (β = −0.120, 95%CI [−0.197, −0.042], p = 0.003),while the negative association between GDF15 and mtDNAcn did not reach significance.

**Table 2:** Associations of GDF15 with LTL and mtDNAcn.

	LTL				mtDNAcn			
	β1(95%CI)	P1	β2(95%CI)	P2	β1(95%CI)	P1	β2(95%CI)	P2
Total	-.140(-.209,-.071)	<0.001*	-.120(-.197,-.042)	.003*	-.091(-.160,-.022)	.010*	-.032(-.107,.042)	.395
Women	-.170(-.258,-.082)	<0.001*	-.139(-.236,-.043)	.005*	-.129(-.217,-.040)	.005*	-.053(-.144,.038)	.250
Men	-.069(-.180,.041)	.218	-.111(-.248,.026)	.113	-.017(-.129,.094)	.758	-.001(-.140,.138)	.991
Age＜65	-.105(-.201,-.009)	.033*	-.107(-.211,-.003)	.044*	-.158(-.253,-.063)	.001*	-.103(-.199,-.007)	.035*
Age≥65	-.139(-.248,-.030)	.013*	-.129(-.250,-.008)	.037*	.046(-.068,.160)	.424	.110(-.015,.235)	.084
BMI＜24	-.104(-.221,.012)	.079	-.034(-.172,.105)	.625	-.218(-.373,-.063)	.006*	-.101(-.270,.067)	.238
24 ≤ BMI＜28	-.187(-.303,-.070)	.002*	-.203(-.336,-.070)	.003*	.003(-.095,.100)	.956	-.006(-.114,.102)	.915
BMI ≥ 28	-.126(-.257,.004)	.058	-.104(-.249,.041)	.161	-.063(-.174,.048)	.262	-.012(-.133,.109)	.845
NGT	.043(-.092,.177)	.532	.061(-.085,.202)	.421	-.096(-.223,.031)	.138	-.076(-.213,.060)	.277
Pre-DM/DM	-.199(-.280,-.117)	<0.001*	-.182(-.277,-.087)	<0.001*	-.060(-.144,.025)	.166	-.016(-.106,.078)	.738

BMI:body mass index;NGT:normal glucose tolerance;DM:diabetes mellitus;GDF15:growth differentiation factor 15;LTL:leukocyte lelomere length;mtDNAcn:mitochondrial DNA copy number;

^a^β1 and P1 were unadjusted.

^b^β2 and P2 were adjusted for age,sex,BMI,WC,SBP,TG,HDL-C,UA and HbA1c

* p < 0.05 indicates significance.

In the stratified analyses,the negative associations between GDF15 and LTL were more prominent in subjects of women, overweight or with abnormal glucose tolerance, but similar results were observed in younger and older subjects(Table 2). Moreover, the negative association between GDF15 and mtDNAcn reached significance in subjects with age younger than 65 years (β = −0.103, 95%CI [−0.199, −0.007], p = 0.035) (Table 2**)**.

**Fig. 2:**
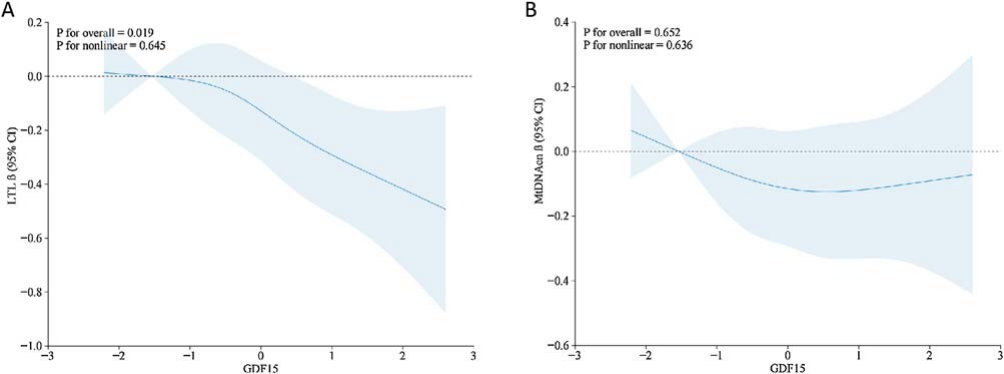
Restricted cubic spline curves between GDF 15 and LTL and mtDNAcn. (A)GDF15-LTL; (B)GDF15-mtDNAcn.

The associations between GDF15 on a continuous scale and LTL were linear in all subjects (p for overall = 0.019,and p for nonlinear = 0.645)and stratified analyses in women,overweight and subjects with abnormal glucose tolerance(Fig. 2, eFig. 1 and eFig. 2). The negative association between GDF15 on a continuous scale and mtDNAcn in all subjects and stratified analyses were not significant(Fig. 2).

## 5 Discussion

This study aimed to investigate the relationship between serum GDF15 and LTL as well as mtDNAcn. After adjusting for confounding factors, we found a significant negative linear association between GDF15 and LTL, which was more prominent in women, overweight individuals, or subjects with abnormal glucose tolerance (AGT). However, the negative association between GDF15 and mtDNAcn did not reach significance.

Previous studies have shown that GDF15 is associated with aging and disease outcomes. Serum GDF15 levels serve as a predictor of all-cause mortality in both a Swedish male cohort and an independent Swedish twin cohort [23]. GDF15 has also been demonstrated to be a robust predictor of overall mortality, as well as cardiovascular and non-cardiovascular mortality, among community-residing elderly individuals from Rancho Bernardo in Southern California [24]. As an emerging marker of aging, few studies have explored the relationship between GDF15 and the classic marker of aging, telomere length. The only study including 120 healthy male individuals found significant linear and negative relationship of GDF15 with telomere length, which was consistent with our study [11]. Aging is a multifaceted process involving numerous molecular pathways. Our study adds another layer to this complexity by suggesting that GDF15 and telomeres are interconnected components within this intricate network, which suggested that the impact of GDF15 might be mediated through telomere dynamics.

Notably, we have observed significant variability in this association across different subgroups, particularly among women, overweight individuals, and those with abnormal glucose tolerance. To gain a deeper understanding of this phenomenon, it is crucial to provide a more detailed interpretation of the underlying mechanisms. Firstly, women exhibit significant hormonal differences compared to men, such as estrogen and progesterone levels. These hormones may influence cellular metabolism and repair mechanisms, thereby modulating changes in telomere length and GDF15 [25]. Secondly,overweight individuals often experience a degree of inflammation [26]. GDF15, with its anti-inflammatory properties, may undergo changes in expression in this context [27]. These changes may impact cellular stress responses and repair mechanisms, thereby enhancing its association with LTL in overweight individuals. Thirdly, individuals with abnormal glucose tolerance often exhibit insulin resistance and produce more oxidative stress products, leading to decreased cellular glucose utilization and increased risk of cellular damage and telomere shortening. GDF15 may regulate insulin sensitivity and cellular metabolism [28], thereby having a more significant impact on telomere length in this subgroup.These findings may provide new insights into understanding the impact of GDF15 on the aging process across different populations. Future studies can further explore the mechanisms underlying these differences and the specific roles of GDF15 under different physiological and pathological conditions.

GDF15 serves as a valuable diagnostic marker for mitochondrial diseases indicating its potential as a biomarker for mitochondrial dysfunction [29]. However, the association between GDF15 and mtDNAcn in our study did not reach significance. The lack of statistical significance in the association can be explored through several potential biological mechanisms and technical limitations. Firstly,mitochondria within a cell can exhibit heterogeneity [30]. This variability can mask any consistent association between a particular phenotype and mtDNAcn, especially if the sampling method does not adequately capture this heterogeneity.Secondly,mtDNAcn can differ significantly across tissues [31], the mitochondrial DNA content in peripheral blood may not necessarily reflect the very aging process. Thirdly, small sample sizes can limit the power to detect significant associations, especially if the effect size is modest. Finally, the regulation of mtDNA copy number is complex and involves multiple genetic and environmental factors. Therefore, it is unlikely that a single factor, such as GDF15, would be solely responsible for changes in mtDNA copy number.Future research can further investigate the relationship between GDF15 and mtDNAcn by expanding sample sizes, optimizing experimental methods, and other approaches.

### 5.1 Strengths and limitations

The study has some strengths: Firstly, by performing subgroup analysis, the study was able to detect interesting differences in the association between GDF15 and LTL across different populations, such as women and overweight individuals. This adds depth to the understanding of how GDF15 might affect aging in different groups. Secondly, given the increasing focus on understanding the aging process and developing effective strategies for prevention and treatment of age-related diseases, the findings of this study contribute significantly to the field of aging research.

The study also has some limitations: Firstly, the study employed a cross-sectional design, which means it can only establish associations between variables but not causal relationships. Longitudinal studies would be more suitable to determine the directionality of these associations. Secondly, although the study adjusted for some confounding factors, there may be other unmeasured or unknown variables that could have influenced the observed associations. Also, the study were lack of clinical assessment of aging process, such as frailty. Lastly, the sample size of this study is somewhat limited, necessitating further studies with larger sample sizes to better elucidate this relationship. However, in comparison to existing research, our study boasts a relatively larger sample size, which contributes to the robustness of our findings.

## 6 Conclusions

In conclusion, this study revealed a negative linear association between GDF15 and LTL and preliminarily explored the differences in this association across different populations. These findings provide important clues for our deep understanding of the role of GDF15 in the aging process and offer new potential targets for the prevention and treatment of aging-related diseases. However, further in-depth research is still needed to fully reveal the specific role of GDF15 in aging mechanisms and its relationship with other aging biomarkers.

### 6.1 Authors' contributions

JY conducted research, performed statistical analysis, and wrote paper. YW, HBZ, FP and WL, HBZ, FP, conducted research. LLX and YXL: designed research and had primary responsibility for final content. All authors have read and approved the manuscript for publication.

## Funding

This research is supported by National High Level Hospital Clinical Research Funding (2022-PUMCH-B-015) and National Natural Science Foundation of China (Grant No.82100947).

## Competing interests

The authors declare that there is not any conflict of interest.
