# Supplementary material for: Serum Growth Differentiation Factor 15 is Negatively Associated with Leukocyte Telomere Length
Source: J Nutr Health Aging. 2025 Feb 3;29(4):100493. doi: 10.1016/j.jnha.2025.100493 (PMC12179997; doi:10.1016/j.jnha.2025.100493)
Supplement: Supplementary file 1 [file mmc1.docx]

**
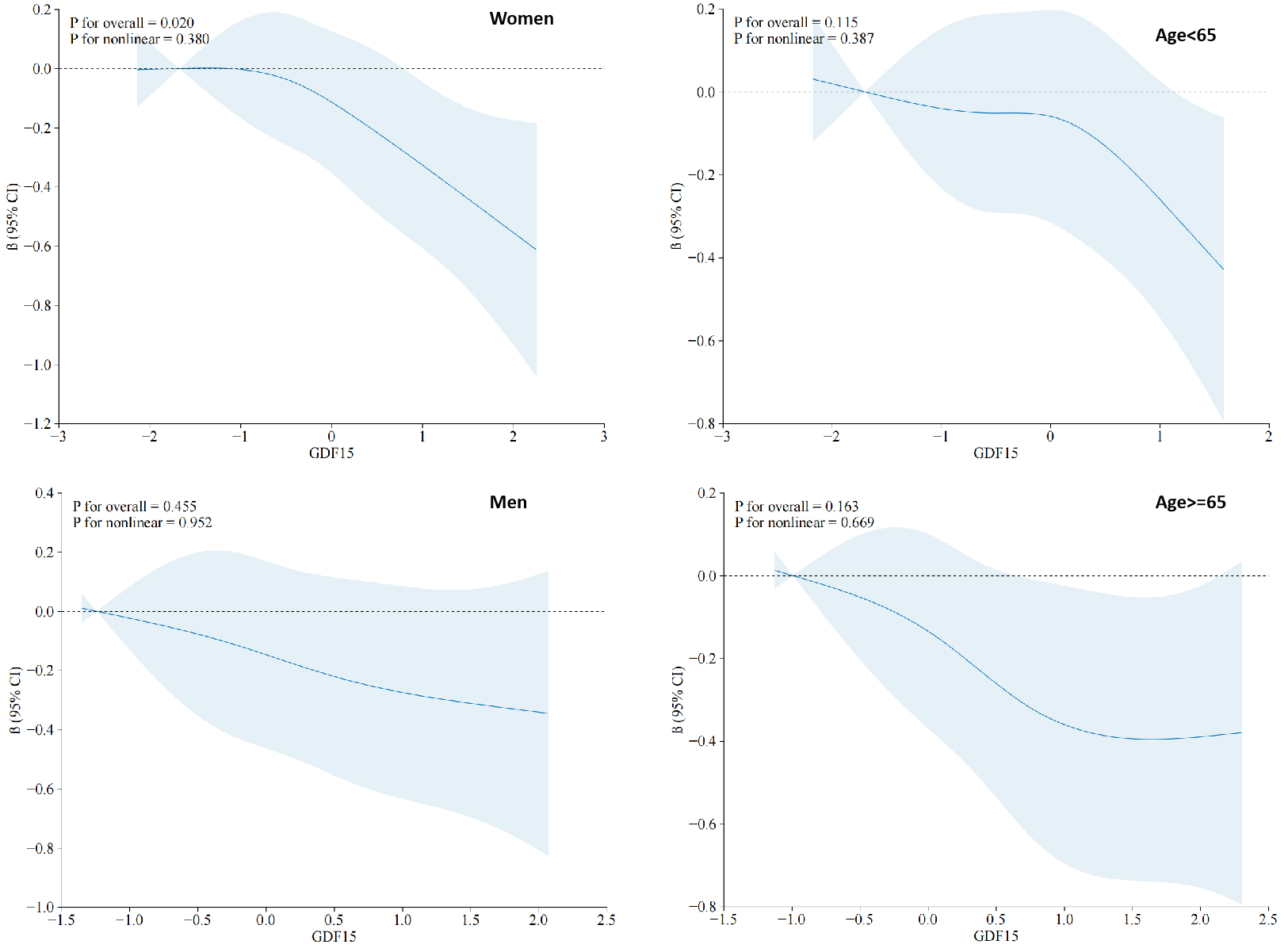
**

**eFigure 1** Restricted cubic spline curves between GDF 15 and LTL in stratified analyses by age and sex.

**
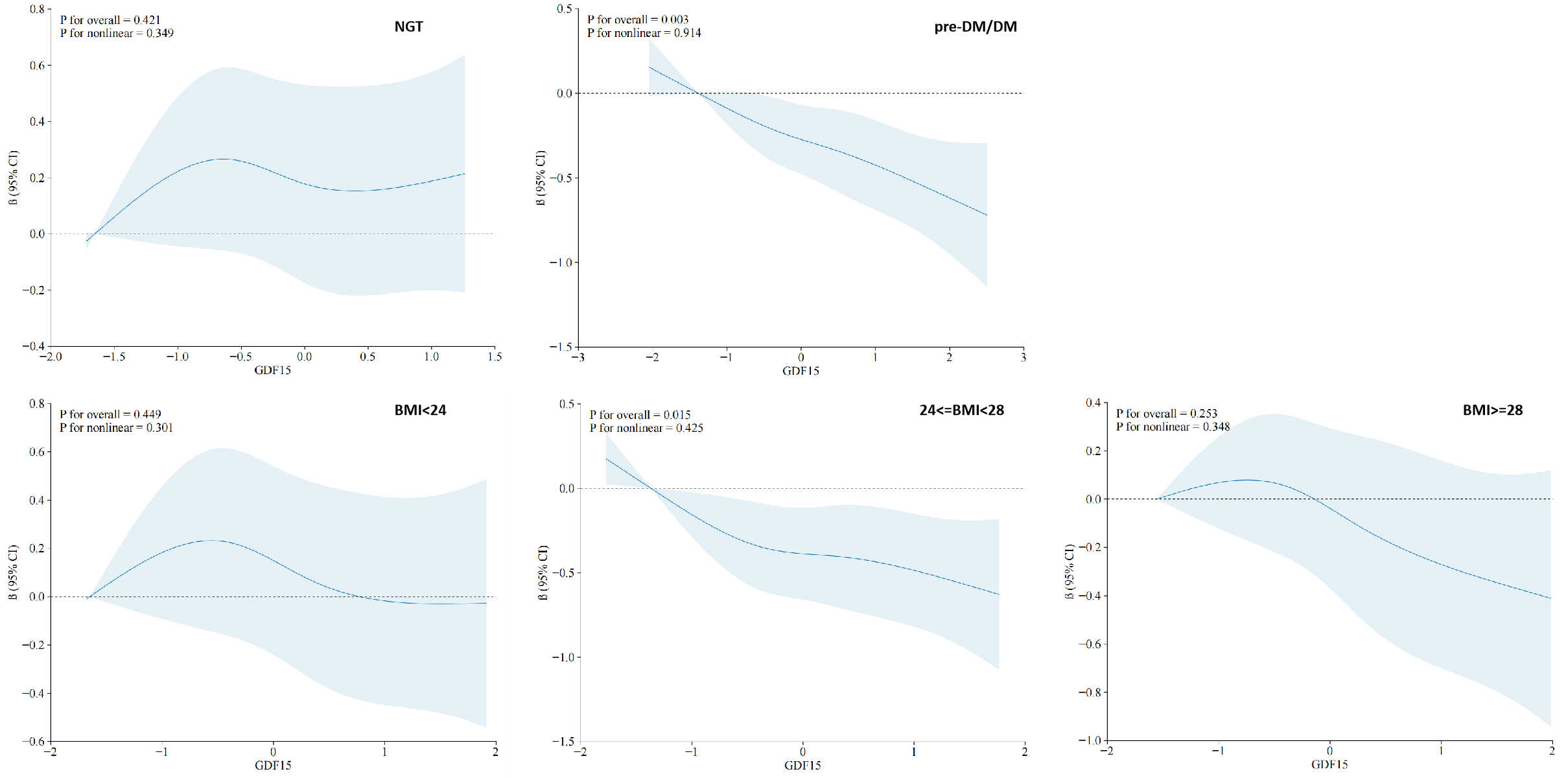
**

**eFigure 2** Restricted cubic spline curves between GDF 15 and LTL in stratified analyses by BMI and glucose tolerance status.
